# Contextual fear conditioning leads to hypo-innervation of the left ventricular myocardium in female but not male C57BL/6J mice

**DOI:** 10.1016/j.ibneur.2026.04.014

**Published:** 2026-05-01

**Authors:** Luca M. Lautenschläger, Lara Mariel Chirich Barreira, Jonas Labode, Julia Schipke, Anne Albrecht, Christian Mühlfeld

**Affiliations:** a Hannover Medical School, Institute of Functional and Applied Anatomy, Carl-Neuberg-Straße 1, Hannover 30625, Germany; b Institute of Anatomy, Otto-von-Guericke-University, Leipziger Straße 44, Magdeburg 39120, Germany; c Center for Behavioral Brain Sciences (CBBS), Universitätsplatz 2, Magdeburg 39106, Germany; d Biomedical Research in Endstage and Obstructive Lung Disease Hannover, Member of the German Center for Lung Research (DZL), Hannover, Germany; e German Center for Mental Health (DZPG), partner site Halle-Jena-Magdeburg, Germany

**Keywords:** Contextual fear conditioning, Autonomic nervous system, Design-based stereology, Heart innervation, Hypo-innervation, Sex differences

## Abstract

**Objective:**

Contextual fear conditioning is an effective way of inducing and modelling anxiety-like states in rodents like mice. We examined the sex-dependent effects of contextual fear conditioning on the autonomic innervation of the left ventricular myocardium in female and male mice.

**Methods:**

Contextual fear conditioning was induced in male and female C57BL/6J mice and compared to male and female controls (n = 5 each). The experimental procedure consisted of two days of habituation, one day of training and one day of context fear memory retrieval. 24 h after context fear memory retrieval, hearts were harvested. PGP9.5-staining was performed to visualize autonomic innervation of left ventricular myocardium. Design-based stereology was used to quantify nerve fibre length density and total length at light microscopic and cardiomyocyte ultrastructure at electron microscopic level.

**Results:**

Contextual fear conditioning led to a hypo-innervation of the left ventricular myocardium in female but not male mice, implicating a sex-dependent autonomic response to and integration of stressful stimuli. Cardiomyocyte ultrastructure was not significantly affected by fear conditioning in both sexes.

**Conclusion:**

Contextual fear conditioning had a profound morphological effect on the innervation of the left ventricular myocardium of female but not male mice. This may have implications for cardiac function in female patients with anxiety disorder and requires further investigation.

## Highlights

• Contextual fear conditioning can alter myocardial nerve fibre length and density.
• Only female mice show myocardial hypo-innervation.
• Autonomic stress integration might favour pro-arrhythmogenic states in females.

## 1 Introduction

In virtually all higher animals, fear and anxiety-like behaviours increase the survival of the individual by triggering stereotypical protective behaviour to potentially aversive external stimuli (Tovote et al., 2015). Despite this significant function, dysregulation of fear responses, seen for example in anxiety disorders (AD), poses a health burden with a lifetime prevalence of up to ∼ 34% (Kessler et al., 2012). For women, the chance of developing an AD is about twice as high, as it is for men (Bandelow and Michaelis, 2015). Therefore, studies investigating the formation of fear and anxiety are crucial for understanding their consequences for the health of the individual, especially focussing on differences between the sexes. The mechanisms of fear formation are well conserved within mammals (reviewed in LeDoux, 2012), and thus can be effectively modelled in rodents like mice. For the study of fear formation, several paradigms are used, most of them being variations of Pavlovian fear condition, where, an aversive stimulus (e.g. an electric foot shock) is used as the unconditioned stimulus (US). The US is simultaneously presented with a conditioned stimulus (CS) that is a neutral stimulus, which is not innately triggering a fear response (e.g. a tone, an odour or a context). After a subsequently paired protocol, the CS is associated with the US, triggering a fear response of the animal, when confronted with the CS, even in absence of the US (Maren, 2001). In contextual fear conditioning (CFC), the CS is not a tone or an odour, but the context, the conditioning procedure takes place in, that is associated with the administration of a foot shock. Several brain regions are involved in subsequent fear memory formation, especially important being the amygdala and the hippocampal formation (Tovote et al., 2015). Behavioural changes following CFC are well described. In rodents, the so-called freezing behaviour is the predominant fear response, i.e. a significant reduction of translational movements, only breathing dependent motions of the ribcage are observed (Fanselow and Ponnusamy, 2008). Studies investigating these behavioural differences reported conflicting results when the sex of the animals tested was considered. Some studies found more freezing behaviour in male (Colon et al., 2018, Kudo et al., 2004, Maren et al., 1994), some in female animals (Keiser et al., 2017, Moore et al., 2010), thereby implicating sex-dependent effects of CFC on fear response. In the last years, increasing interest in sex-specific differences led to an increasing number of studies investigating the differences between males and females, especially concerning changes in brain anatomy and –biochemistry (Keady et al., 2024, Boutros et al., 2023). However, the effects of CFC on the autonomic nervous system (ANS) and the heart are rarely studied, although it is very well known, that CFC leads to an activation of the sympathetic outflow to the heart (Stiedl et al., 2004), whereby increasing heart rate and blood pressure. A (hyper) activation, as might develop as a result of sustained CFC paradigms (Stiedl et al., 2004), of the sympathetic nervous system (SNS), innervating the heart is known to be an important contributor to several heart diseases in humans, favouring disease progression and devastating health outcomes (reviewed in Grassi and Drager, 2024). In a murine α_2A_-/ α_2__C_-adrenoreceptor knockout model, elevated sympathetic tone was observed, leading to a decline in heart function and damaging the myocardial ultrastructure (Brum et al., 2002). This damage could in part be dependent on higher noradrenaline (NA) concentrations, as sustained high plasma NA concentrations could be shown to lead to a sympathetic denervation of the left ventricle in mice (Kimura et al., 2010). As an indicator for sympathetic function within the heart, this study quantified the innervation length and –density of the left ventricle and tested the impact of CFC on left ventricular myocardial ultrastructure, especially left ventricular innervation density, focussing on putative sex-differences.

## 2 Materials and methods

### 2.1 Animals and contextual fear conditioning (CFC)

All animal experiments were in accordance with the local authorities (Landesverwaltungsamt Sachsen-Anhalt, permission number 203.m-42502–2–1717 UniMD), the German animal welfare law and the EU directive 2010/63/EU.

Adult male and female C57BL/6J mice (n = 10 each; 20 animals in total) were housed in the central animal facility of the Otto-von-Guericke-University Magdeburg in groups of three to four animals per cage, under a reverse 12 h: 12 h light: dark cycle with food and water ad libitum. Cages were equipped with plastic houses, tissue, nesting and bedding materials. Lights were turned off from 9 am to 9 pm.

**Fig. 1:**
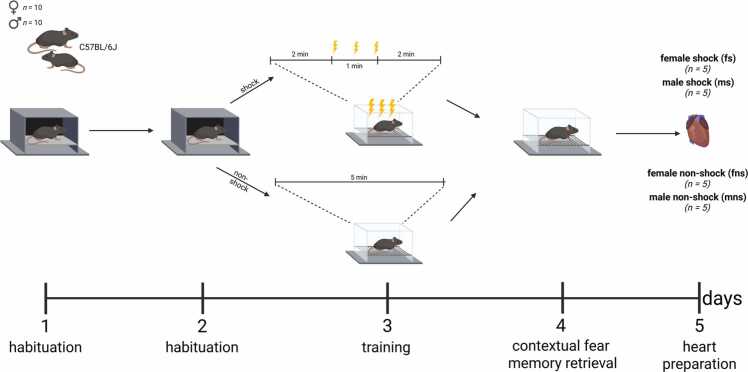
Contextual fear conditioning paradigm. Created in https://BioRender.com.

Before behavioural experiment started, mice were handled for three days, 10 min per cage, to get used to the female investigator (LCB). All experiments were conducted at the mice’s active dark phase by the same female investigator (LCB) between 10 am and 12 pm. The CFC experiment was conducted on four consecutive days and consisted of two days of habituation, one day of training and one day of context fear memory retrieval (Fig. 1). All procedures were performed using a fear conditioning system that consisted of an acoustically isolated box, equipped with a fan (Ugo Basile, Gemonio, Italy; 46101/46101–2/46101–3/46101–4). The box measured 250 × 250 × 360 × mm (*w* x *d* x *h*), contained interchangeable wall panels and floor surfaces, enabling the establishments of different contexts. In the first two days of the CFC paradigm, habituation took place, where mice could freely explore a neutral context for 6 min per day. Neutral context consisted of a black and white striped wall on the left, a black and white checked wall on the right and a black wall in the back, with a grey floor inside the fear conditioning cubicle. An 0.2% acetic acid solution was used to clean the boxes before and after each habituation and also served as an olfactory cue.

After habituation, mice were randomly assigned into two groups: shock and non-shock. Both groups consisted of mice of both sexes, resulting in the following design: female non-shock (fns), female shock (fs), male non-shock (mns) and male shock (ms), with n = 5 animals per subgroup.

During training on day three, animals were exposed to the shock-context, consisting of transparent walls and a grid metal floor, as well as 70% ethanol as olfactory cue (Fig. 1). Control animals (fns, mns) were allowed to explore the box freely for 5 min without shock exposure, while animals in the shock-exposure group (fs, ms) explored the box for 2 min (pre-shock) before three foot-shocks were delivered as an aversive, i.e. unconditioned stimulus (US; strength: 0.4 mA, duration: 1 s each, at 20 s intervals). After shock exposure, mice stayed in the box for additional 2 min (post-shock phase).

Long-term contextual fear memory was tested during the retrieval phase, 24 h after training. During retrieval, mice were transferred into the shock-context again (without shock delivery), and animals could explore freely for 6 min.

Within each trial, movement of each animal was recorded by EthoVision® XT and subsequently analysed using the EthoVision® XT 17 software (Noldus, Wageningen, the Netherlands). Context fear was analysed by freezing, the time that animals were immobile during the first 2 min of the retrieval. Freezing levels were automatically determined by the video tracking software as an immobility score < 4% (less than 4% change in the pixels covering the mouse body between consecutive video frames).

### 2.2 Sample preparation

24 h after context fear memory retrieval, mice were deeply anesthetized with intraperitoneal injections of 250 mg/kg body weight ketamine and 20 mg/kg of body weight xylazine (Sigma-Aldrich, St. Louis, MO, USA). Mice were killed by cervical dislocation. The thoracic cage was opened, the heart was excised and immersion fixed in 4% paraformaldehyde (PFA) (Merck Millipore, Billerica, MA, USA) in 0.2 M phosphate buffer at pH 7.4. Hearts were stored in fixing solution for 24 h at 4 °C, thereafter transferred to phosphate buffer (PO_4_) and stored at 4 °C until further processing.

#### 2.2.1 Heart sampling

The atria were cut off and the left ventricle including the interventricular septum was separated from the right ventricle.

Left ventricles were washed, dried and carefully weighed. Weight of left ventricles was divided by the known density of mammalian muscle tissue (1.06 g/cm^3^) (Méndez and Keys, 1960), to obtain the volume of the samples (containing space), necessary for stereological assessment. Left ventricles were sampled using the orientator (Mattfeldt et al., 1990), to obtain isotropic uniform random (IUR) samples for light microscopy (LM) and transmission electron microscopy (TEM) (Mühlfeld et al., 2010a). When length(-density) should be estimated, a special sampling regime is necessary to ensure isotropy of the structure that is to be measured (i.e. the structure-estimate is then independent of the sample’s orientation). The separated left ventricles are placed on a φ clock that is divided by lines, denoting 5° distances, ranging from 0° (equator) to 99°. Here, the long axis of the left ventricle is placed along the equator of the circle. A random number between one and 99 is chosen and the sample cut accordingly. The cut surface is then placed on a cosine-weighted ϑ clock. Here another random number is chosen, and the sample cut accordingly. For in depth description and depiction refer to (Mattfeldt et al., 1990, Mühlfeld et al., 2010a).

#### 2.2.2 Hearts – light microscopy and immuno-histochemistry

Left ventricle samples for light microscopy were dehydrated by a standard procedure of ascending alcohol series using the MTP carousel tissue processor (Slee, Nieder-Olm, Germany), and embedded in Paraplast Plus (Carl Roth, Karlsruhe, Germany; X881.1) following a standard protocol. Subsequently 5 µm thick sections were cut and stained with an antibody against protein gene product (PGP)9.5 (US Biological, Salem, MA, USA; P9102–70G), a pan-neuronal marker, for detection of nerve fibres within myocardial tissue, as described by (Mierswa et al., 2025). In brief, after rehydration with a descending alcohol series, antigen retrieval was done by microwaving the slices in Dako Target Retrieval Solution, pH 9 (Agilent Technologies, Santa Clara, CA; USA). After cooling for 30 min, hydrogen peroxide (Santa Cruz Biotechnology, Dallas, TX, USA; CAS 7722–84–1) was applied for 5 min to block endogenous peroxidase activity. Avidin- and biotin-blockage (Vector Laboratories, Newark, CA, USA; VEC-SP-2001) was performed for 15 min each. For blockage of unspecific binding-sites, 5% goat serum in PBS with 1% bovine serum albumin and 0.3% TRITON™ X-100 (Sigma-Aldrich, St. Louis, MO, USA; T9284) was applied before incubation with the primary rabbit antibody against PGP9.5 (dilution 1:500) overnight at 4 °C. Then a secondary goat anti rabbit biotinylated antibody (Dianova, BIOZOL, Hamburg, Germany; Sec-183348) was applied and incubated for 60 min. Thereafter an avidin-biotin complex (ABC) vector kit (VECTASTAIN® Elite® ABC-HRP-Kit) (Vector Laboratories, Newark, CA, USA; PK 6100) was used and incubated for 45 min. 3–3’-diaminobenzidine (DAB) (Vector Laboratories, Newark, CA, USA; SK-4100) was added. Enzymatic reaction was stopped, when sufficient labelling was observed. Hemalaun counterstaining was performed, slices were dehydrated in an ascending alcohol series and covered with a coverslip using ROTI® Histokitt II (Carl Roth, Karlsruhe, Germany; T160.2). If not stated otherwise, all procedures were performed at room temperature.

#### 2.2.3 Hearts – transmission electron microscopy preparation

For TEM-preparation, heart samples were incubated in 1.5% PFA and 1.5% glutaraldehyde in 0.15 M HEPES buffer (pH 7.35) (Merck Millipore, Billerica, MA, USA) for further fixation. Post-fixation was performed by incubating the samples in 1% osmium tetroxide (OsO_4_).

Samples were contrasted, using 1% uranyl acetate and then dehydrated in an ascending acetone series (70%, 90% and 100%). Samples were then embedded in epoxy resin (Serva Electrophoresis, Heidelberg, Germany), and 60 nm thick sections were cut and collected on Formvar (em-grade, Mauressac, France) coated copper grids. Sections were contrasted with 4% uranyl acetate and lead citrate.

#### 2.2.4 Transmission electron microscopy

TEM was performed using a Morgagni 268 transmission electron microscope (FEI, Eindhoven, the Netherlands) equipped with a side mounted Veleta digital camera (Olympus Soft Imaging Solutions, Münster, Germany). Pictures were taken, following systematic uniform random sampling (SURS) principles (Gundersen and Jensen, 1987). Counting was carried out by using the STEPanizer software version 1.0 (Tschanz et al., 2011).

#### 2.2.5 Hearts – western blot

Protein isolation from formalin fixed left ventricle samples was performed using the Qproteome FFPE Tissue Kit (Qiagen, Hilden, Germany) following the manufacturer’s instructions. In brief, samples of the left ventricle were cut and 10 mg were weighed, separated and used for further processing. For protein isolation, Extraction Buffer EXB Plus (Qiagen, Hilden, Germany) and 10% β-mercaptoethanol were added and vortexed. The samples were incubated for 5 min on ice, followed by an incubation at 100 °C for 20 min on a Multi-Therm™ heating block (Benchmark Electronics, Tempe, AZ, USA). At 750 revolutions per minute (RPM), the samples were held at 80 °C for 2 h. After cooling the samples at 4 °C for 1 min, the samples were centrifuged. For protein separation by sodium-dodecyl sulfate polyacrylamide gel electrophoresis (SDS-PAGE), 10 µL of supernatant were loaded on each lane of a 14% concentrated gel. SDS-PAGE ran at 130 V. Separated proteins were transferred to polyvinylidene fluoride (PVDF) membranes (Bio-Rad Laboratories, Hercules, CA, USA) at 400 mA current for 70 min. The membranes were blocked with EveryBlot Blocking Buffer (Bio-Rad Laboratories, Hercules, CA, USA; 12010020) and incubated with the primary rabbit antibody against nerve growth factor (NGF) (26 kDa) (Abcam Limited, Cambridge, UK; ab6199), diluted 1:1.000, at 4 °C overnight. After washing, the membranes were incubated with the secondary antibody, Peroxidase-AffiniPure F(ab')2 Fragment Goat Anti-Rabbit IgG (Dianova, Hamburg, Germany; 111–036–045) (Jackson ImmunoResearch Laboratories, West Grove, PA, USA) in an 1:10.000 dilution for 6 h. For loading control a β-actin primary mouse antibody (Santa Cruz Biotechnology, Dallas, TX, USA; sc47778) (1:1.000 dilution) was applied for 48 h and a donkey anti goat secondary antibody (Santa Cruz Biotechnology, Dallas, TX, USA; sc-2020), 1:10.000 diluted, was applied for 1 h. Membranes were developed with WesternBright™ Peroxide (Advansta, San Jose, CA, USA; R-03025-D10) and imaged with the ChemiDoc™ imager (Bio-Rad Laboratories, Hercules, CA, USA). Assessing of intensity of protein bands was performed using the Image Lab software (Bio-Rad Laboratories, Hercules, CA, USA). NGF signals were normalized to β-actin as loading control and are presented as % of the control group mean of the same membrane.

#### 2.2.6 Stereology

To obtain reliable estimates of structural composition of the left ventricle myocardium, as well as quantification of total nerve fibre length in the left ventricular myocardium, design-based stereology was performed.

#### 2.2.7 Stereology – nerve fibre quantification

For quantification of nerve fibre length within the left ventricle, design-based stereology was applied as previously described by (Mühlfeld, 2010b). Sections of left ventricular myocardium, stained against PGP9.5 were analysed using a Leica DM6000 B (Leica, Wetzlar, Germany) microscope equipped with an Olympus DP72 digital camera (Olympus, Hamburg, Germany) and a computer that used The Visiopharm Integrator System (VIS) (Visiopharm, Hørsholm, Denmark; Version 3.6.5.0). At a 40x magnification, four unbiased counting frames were projected on each field of view, together covering an area of 9083.55 µm^2^. Each counting frame consisted of two exclusion lines and two inclusion lines, the upper right corners were used as a point grid for estimating containing space i.e. heart tissue. The tested areas were generated by SURS. PGP9.5-positive nerve fibre profiles were counted if laying within the counting frame or touching the inclusion lines and not touching the exclusion lines. Sampling fraction was chosen, so for every sample between 100 and 200 counting events occurred.

The length density of any given structure *L*_*V*_*(x)* is estimated by multiplying the profile counts of the structure of interest *Q(x)* per area tested *A*_*T*_ by 2:

$$L_{V}\left(x\right)=\frac{2∙Q\left(x\right)}{A_{T}}=2Q_{A}$$

When counting nerve fibre profiles specifically, the following formula yields $Q_{A}$:

$$Q_{A}=\frac{\sum Q(\mathrm{nf})}{\sum A(\mathrm{ref},\;\mathrm{CF})}$$

Where *Q*_*A*_ equals all PGP9.5-positive nerve fibre profile counts *∑Q(nf),* divided by the sum of all areas, that were reference volume within all counting frames *∑A(ref, CF)*. The length density of nerve fibres within the left ventricle *L*_*V*_*(nf/lv)* is then calculated by multiplying the sum of all PGP9.5-positive nerve fibre profile counts *∑Q(nf)* by 2 and dividing by the sum of points (upper right corner of each counting frame) multiplied by the area covered by them *∑P(cf) x a(p)*:

$$L_{V}\left(\mathrm{nf}/lv\right)=\frac{2∙\sum Q(\mathrm{nf})}{(\sum P(\mathrm{cf})∙a(p))}$$

To obtain absolute measures, this length density needs to be multiplied with the measured containing space, i.e. the volume of the respective left ventricle *V(lv)*:

$$L\left(\mathrm{nf},\;lv\right)=\frac{\sum Q(\mathrm{nf})}{\left(\sum P(\mathrm{cf})∙a(p)\right)}∙V(lv)$$

#### 2.2.8 Stereology – composition of cardiomyocytes

To estimate the ultrastructural composition of cardiomyocytes, a point grid was superimposed onto EM micrographs. Points were counted if “hitting” the structure investigated. Number of points was chosen in a manner that ensures between 100 and 200 counting events per structure per animal. The number of points hitting the designated structure, e.g., lipid droplets (P_ld_) is then divided by points hitting containing space, i.e., cardiomyocyte (P_cm_). This yields the volume density (V_V_) of the structure investigated (V_V_(ld/cm)). This relative value can then be multiplied by the measured containing space, in this case the volume of cardiomyocytes within the left ventricle (V(cm, lv)). Thereby absolute measures are generated, in this example the absolute volume of lipid droplets within the cardiomyocytes (V(ld, cm)). This procedure can then be repeated for all structures for which quantitative analysis is desired.

#### 2.2.9 Statistics

All statistical analyses were performed using R (version 4.5.1) (R Core Team, 2025). Since five animals per group do not suffice to evaluate normality of residuals, nor homoscedasticity, for statistical evaluation with two independent variables (sex and group, i.e. “shock” and “non-shock”), Scheirer-Ray-Hare test and Dunn’s post hoc test were performed, using the “FSA”- and “rcompanion”- packages (Mangiafico, 2025, Ogle et al., 2025). Results were considered statistically significant if p ≤ 0.05. Visualisation of data was performed with GraphPad Prism software (version 9.2.0) (GraphPad Software, Boston, MA, USA). All graphs show median values and interquartile ranges (IQR). Stars indicate the following statistical significances: * = p ≤ 0.05; ** = p ≤ 0.01; *** = p ≤ 0.001.

## 3 Results

**Fig. 2:**
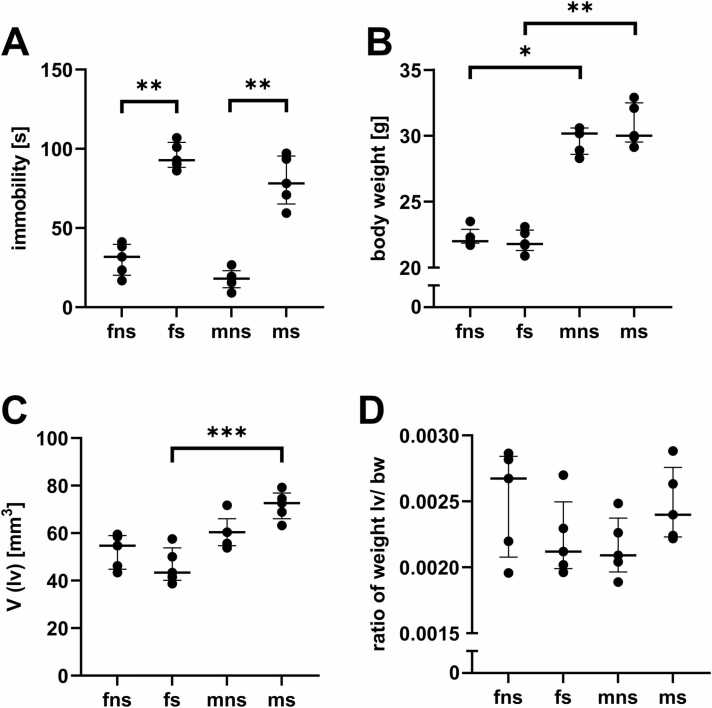
Freezing behaviour and weight metrics. (A) Measured time, the animals spent freezing (immobility score less than 4%) at the first 2 min of context fear memory retrieval. (B) Body weight of the animals, measured at the day the organs were harvested. (C) Volume of the left ventricle that was calculated using the known density of mammalian muscle tissue. (D) The ratio of body weight to left ventricular weight. All graphs show median values with IQR.

**Fig. 3:**
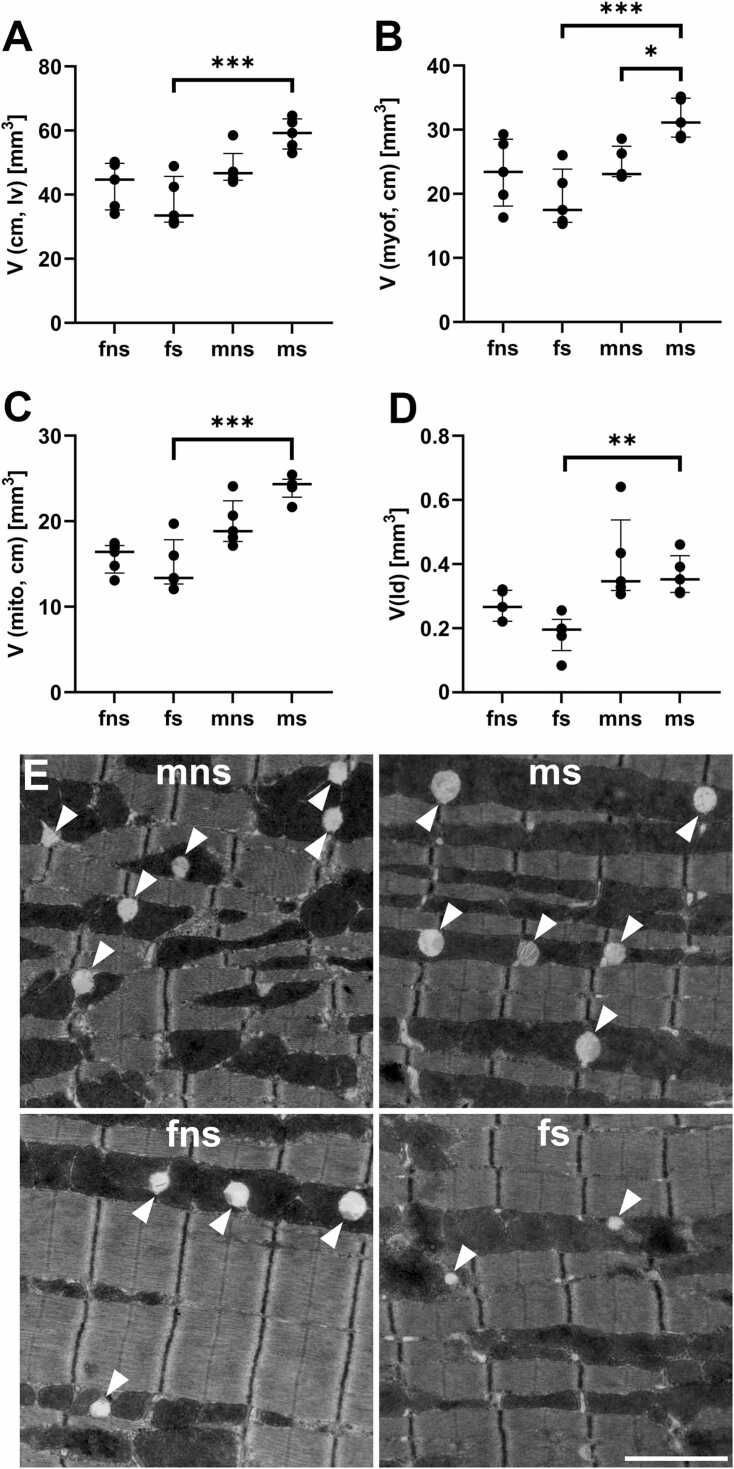
Stereological parameters of cardiomyocyte ultrastructure. (A) Volume of cardiomyocytes within the left ventricular tissue. (B) Volume of myofibrils within the cardiomyocytes of the left ventricular tissue. (C) Volume of mitochondria within the cardiomyocytes of the left ventricular tissue. (D) Volume of lipid droplets within the cardiomyocytes of the left ventricular myocardium. (E) Representative electron micrographs, arrowheads indicating lipid droplets. mns: male non-shock, ms: male shock, fns: female non-shock, fs: female shock. Scale bar: 2 µm. All graphs show median values with IQR.

**Fig. 4:**
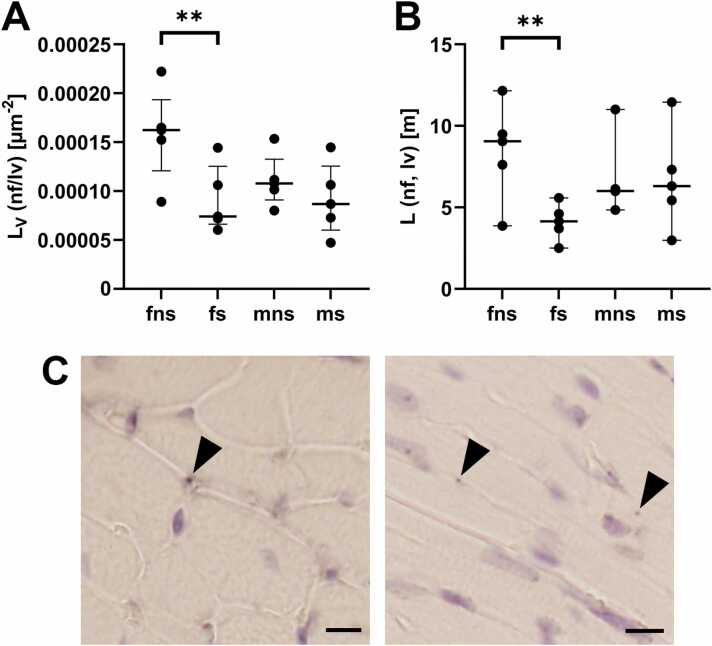
Nerve fibre length and -length density. (A) PGP9.5-positive nerve fibre length density within the left ventricular myocardium. (B) Absolute PGP9.5-positive nerve fibre length. (C) Representative micrographs showing PGP9.5-positive nerve fibres (arrowheads). Note the position between the cardiomyocytes. Scale bar: 15 µm. All graphs show median values with IQR.

**Fig. 5:**
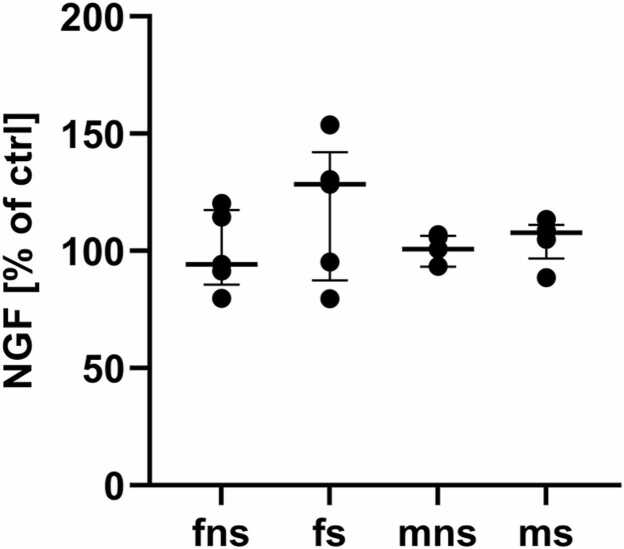
Western blot results for NGF-concentration within the left ventricular myocardium. The graph shows median values with IQR.

When comparing the freezing behaviour at contextual fear memory retrieval, group had a significant influence on the time spent freezing (p = 0.00016) (Fig. 2A). Both male (p = 0.005) and female mice (p = 0.01), that received shocks, froze significantly more, than their respective controls. No such effect was present, when comparing fns and mns (p = 0.36) or fs and ms (p = 0.49). Statistical testing showed a significant effect of sex on the body weight of the animals (p = 0.00015), with male mice being heavier than female mice (Fig. 2B). Group had no significant effect (p = 0.97) on the body weight of the animals. The volume of the left ventricle was significantly dependent on the sex of the animal (p = 0.001) (Fig. 2C). When comparing the groups individually, no difference between the left ventricular volume of fns and mns was present (p = 0.27) but between fs and ms with male mice having a greater left ventricular volume (p = 0.0005). The left ventricle-to-body weight ratio revealed no statistical difference between the groups (Fig. 2D). To take this into consideration, all stereological parameters were also expressed relative to body weight (Table 1). The volume of cardiomyocytes within the left ventricle showed significant dependence on sex (p = 0.005), as well as on the interaction between sex and group (p = 0.041) (Fig. 3A). Post hoc test showed that this was primarily due to the significant difference between fs and ms (p = 0.0006). Relative to body weight, the volume of cardiomyocytes showed no significant dependence on group (p = 0.82) or sex (p = 0.82) but on their interaction (p = 0.019) (Table 1). The volume of myofibrils within these cardiomyocytes was significantly dependent on sex and the interaction between sex and group (p = 0.01) and (p = 0.019), respectively (Fig. 3B). ms mice had significantly higher volume of myofibrils per cardiomyocytes than their control (p = 0.04). fs showed significantly lower volume of myofibrils per cardiomyocytes than ms animals (p = 0.0006). This significant dependence was abolished, when computed relative to body weight. Only the interaction between sex and group remained significantly different (p = 0.02). The volume of mitochondria per cardiomyocytes showed significant dependence on sex as well (p = 0.0005) (Fig. 3C). Here as well, fs animals had significantly lower volumes than ms (p = 0.001), whilst no such difference was detectable in control animals (p = 0.0975). Here, no dependence on the interaction of sex and group could be detected (p = 0.257), neither when computed relative to body weight (Table 1). The volume of lipid droplets within the cardiomyocytes was significantly dependent on sex (p = 0.00088) (Fig. 3D). No significant difference could be detected between mns and fns (p = 0.087), but between fs and ms (p = 0.0028). This effect could also be shown, when the volume of lipid droplets was related to body weight, where group and sex showed tendency to significance (p = 0.059 and p = 0.07) and post-hoc test showed significant differences between fns and fs (p = 0.028), and fs and ms (p = 0.033). No significant difference could be detected between fns and mns (p = 0.669). When comparing the length density of PGP9.5 positive fibres within the left ventricle, significant dependence could be shown for group (p = 0.01), that was due to the significant difference between fns and fs, where fs showed significantly lower density (p = 0.01) (Fig. 4A). When the length density of PGP9.5 positive nerve fibres was computed relative to body weight and statistical testing was performed, the length density showed statistically significant dependence on sex (p = 0.034) and group (p = 0.028). Post-hoc testing showed, that fns had a significantly higher density, compared to fs (p = 0.037) as well as to mns (p = 0.042). For total nerve fibre length, group showed a strong tendency to significance (p = 0.059). Comparison between groups showed a significantly lower total nerve fibre length in fns, compared to fs (p = 0.01) (Fig. 4B), that could be shown as well, when computing relative to body weight (p = 0.037). The NGF amount within the left ventricle showed neither a significant dependence on group, factor or their interaction, nor did any pairwise comparison show a significant difference (Fig. 5).

**Table 1:** Stereological parameters of cardiomyocyte ultrastructure in relation to body weight.

	fns	fs	mns	ms
V(lv) [mm^3^]	52.45 ± 6.50	46.23 ± 6.78	60.38 ± 6.23	71.70 ± 5.40
V(lv) / bw [mm³ /g]	2.36 ± 0.34	2.09 ± 0.25	2.03 ± 0.19	2.33 ± 0.24
V(cm, lv) [mm^3^]	42.94 ± 6.61	37.54 ± 6.997	48.28 ± 5.22	58.97 ± 4.33
V(cm, lv) / bw [mm³ /g]	1.93 ± 0.33	1.698 ± 0.27	1.62 ± 0.16	1.92 ± 0.1998
V(myof, cm) [mm^3^]	23.32 ± 4.82	19.26 ± 4.06	24.66 ± 2.38	31.74 ± 2.74
V(myof, cm) / bw [mm³ /g]	1.05 ± 0.23	0.87 ± 0.16	0.83 ± 0.08	1.03 ± 0.12
V(mito, cm) [mm^3^]	15.73 ± 1.58	14.87 ± 2.74	19.77 ± 2.45	23.96 ± 1.24
V(mito, cm) / bw [mm³ /g]	0.71 ± 0.08	0.67 ± 0.11	0.66 ± 0.07	0.78 ± 0.06
V(ld, cm) [mm^3^]	0.27 ± 0.04	0.18 ± 0.06	0.41 ± 0.12	0.37 ± 0.06
V(ld, cm) / bw [mm³ /g]	0.0120 ± 0.0018	0.0082 ± 0.0023	0.0138 ± 0.0038	0.0119 ± 0.00198
L_V_(nf/lv) [10^−5^ x µm^−2^]	15.8 ± 4.23	9.14 ± 3.05	11.1 ± 2.39	9.16 ± 3.28
L_V_(nf/lv) / bw [10^−6^ x µm^−2^/g]	7.15 ± 2.05	4.19 ± 1.54	3.73 ± 0.76	2.98 ± 1.13
L(nf, lv) [m]	8.44 ± 2.71	4.11 ± 1.02	6.8 ± 2.16	6.70 ± 2.78
L(nf, lv) / bw [m/g]	0.38 ± 0.13	0.19 ± 0.05	0.23 ± 0.07	0.22 ± 0.096

All values are given as mean plus/minus standard deviation. mns: male non-shock, ms: male shock, fns: female non-shock, fs: female shock.

## 4 Discussion

The present study showed that a CFC paradigm, consisting of a strong, yet temporarily acute event, was sufficient for contextual fear formation, seen as more time spent freezing. The most important finding of this study is, that independent of behavioural differences, neuroanatomical changes of the autonomic innervation of the left ventricular myocardium were observed in a sex-dependent manner. The quantitative morphological (stereological) assessment of PGP9.5-positive nerve fibres within the myocardium of the left ventricle revealed, that hypo-innervation, i.e. a decrease in total nerve fibre length and length density of PGP9.5-positive nerve fibres, was only present in female mice that had previously received shocks, when compared to respective non-shocked controls. No such effect was present, when analysing male mice.

This study methodologically relies on earlier work by Mühlfeld and colleagues that established an unbiased (design-based) stereological approach to quantify total nerve fibre length and –density, as well as total axonal length (Mühlfeld, 2010b). This method was used in a slightly simplified manner, by not quantifying the mean number of axons per nerve fibre, making it impossible to answer the question of total axonal length. This parameter did not seem to be of the uttermost importance, since a decrease in length density (L_V_) of PGP9.5-positive nerve fibres, indicates that an average nerve fibre innervates a larger area of myocardial tissue, irrespective of a potentially changed mean axonal number. This method, even in its simplified form, provides biologically meaningful data that is more precise, than semi-quantitative methods and meets all sampling criteria of design-based stereology, ensuring unbiased estimates. Despite this method being applicable for the right ventricle as well (Mierswa et al., 2025), the present study focussed on the left ventricle because of its functionally central role in supplying all of the organism’s tissue with oxygenated blood. Statistical analysis revealed significant differences between the volumes of the left ventricles of fs and ms. The ratio of left ventricle and body weight revealed no such difference. Since the volume of the left ventricle acts as the containing space for other parameters, almost all myocardial parameters were significantly different between fs and ms. Taking this into consideration, all other quantitative (ultra-)structural parameters did not differ between the respective groups indicating that the experimental procedure did not result in alterations of the cardiomyocytes and their composition (Table 1). This was analysed by computing the stereological data in relation to the body weight of the animal, whereby confirming, that no gross alteration in myocardial ultrastructure took place. Interestingly, the volume of lipid droplets, relative to the body weight of the animals was significantly reduced in fs compared to fns and fs to ms (Table 1). This could be a secondary effect of the hypo-innervation or a response of the cardiomyocyte itself to a strong autonomic activation due to the fear conditioning. There was no morphological evidence of an acute injury of the myocardium.

This study was carried out using n = 5 mice per group and sex as an exploratory morphological study. Despite the small sample size of the subgroups, this study was able to detect changes in autonomic innervation (-density) in female but not male mice, following a moderate yet highly acute, stressful event. This suggests, that the differences in the autonomic reaction to stressful events in female and male mice found here depict relatively strong effects.

NGF, perhaps the most prominent member of the neurotrophin growth factor family, has important functions in establishing myocardial innervation during development and regulating it during adulthood (reviewed in Hasan, 2013). NGF mRNA content is positively correlated to the sympathetic innervation density of several organs and their NA content in various mammalian species (Shelton and Reichardt, 1984). This positive correlation holds also true for NGF mRNA and NGF protein concentrations of the murine heart (Heumann et al., 1984). NGF overexpression in a transgenic mouse model, did not only lead to a hyper-innervation of the heart, but also to a significant increase of cardiac mass, indicating its important role in homeostasis and adaption to changing demands (Hassankhani et al., 1995). We therefore hypothesized, that the observed hypo-innervation was possibly due to a reduced NGF content of the myocardial tissue of female shocked mice, compared to their respective controls. For evaluation of NGF protein content, tissue lysates of left ventricle myocardium were used to perform WB analyses. As reported above, no difference between the experimental groups was present. Two main reasons could explain this finding. Firstly, for stereological purposes the whole heart had to be fixed chemically using 4% PFA which influences WB analyses and may have caused an imprecise measurement and made analysis of NGF mRNA content impossible. Secondly, as shown in earlier work, the change in NGF content of cardiac tissue that led to a quantifiable hypo-innervation, occurred within a longer period of time than reported in this study (Mühlfeld et al., 2011). Even if NGF signalling was involved, the changes after 48 h might be minuscule and masked by imperfect condition of cardiac tissue. This indicates the responsible mechanism to be in part or entirely independent of NGF signalling and is in line with previous work, describing NGF involvement in much brisker settings, such as myocardial infarction, accompanied by massive damage to cardiomyocytes (Zhou et al., 2004) or tumour cachexia and an elongated time line (Mühlfeld et al., 2011). The unaltered NGF content could also indicate that the observed hypo-innervation was not (exclusively) sympathetic in nature. Within porcine hearts as a model, it could be shown that not only the atria are densely innervated by acetylcholine esterase positive fibres, but also the ventricles (Ulphani et al., 2010). Since PGP9.5 is a pan-neuronal marker, in this study, no differentiation could be made between sympathetic, parasympathetic efferent or sensory afferent fibres. The reduction in PGP9.5-positive nerve fibres could therefore be due to a loss in any of the above, or a combination of those. Although parasympathetic efferent fibres reach the ventricles, innervation density of those fibres within the atria exceeds that of the ventricles by up to 5.9 times (Kawano et al., 2003). It is therefore appropriate to state, that the hypo-innervation at least in part was sympathetic in nature: in a model of murine cardiac hypo-innervation, hearts showed blunted response to stellate ganglion stimulation but increased sensitivity to systemically circulating catecholamines, resulting in a pro-arrhythmogenic state (Tapa et al., 2020). Similar effects have been shown to occur in a rabbit model of hypo-innervation following myocardial infarction (MI) (Guevara et al., 2024). This “denervation supersensitivity” for catecholamine (mostly NA) could also be shown in a canine model of chemically induced myocardial denervation (Warner et al., 1993), where it resulted in blunted responses to sympathetic stimulation and increased responses to applied NA, with no changes in beta-adrenoreceptor density nor -affinity. This heterogeneously denervated myocardial tissue is hypothesized to be pro-arrhythmogenic and being the leading cause for post-MI arrhythmia (Rubart and Zipes, 2005). This indicates that the observed hypo-innervation, despite not being caused by a gross damage to the myocardial tissue, may also lead to a pro-arrhythmogenic state that poses substantial risks of ventricular arrhythmias and therefore needs further attention to elucidate the underlying mechanisms of denervation.

This study showed, that an acute CFC paradigm results in contextual fear formation and led to a significant decrease in total PGP9.5-positive nerve fibre length density and total nerve fibre length of left ventricular myocardium in female, but not male C57BL/6J mice left ventricles. This was irrespective of putative behavioural sex differences. With a design-based stereological approach, we were able to show for the first time that the CFC impact occurs subacutely within 48 h after training. These findings might bare clinical relevance, proposing that integration of and reaction to stressful stimuli might occur even in a relatively short time span after stress exposure in a sex-dependent manner, possibly resulting in a state of increased risk for ventricular arrhythmias in female subjects.

These findings add to a growing body of evidence on the systemic integration of aversive, stressful events along the different brain-body axes. During fear conditioning and the retrieval of aversive memories, the short acting sympathetic system and the long lasting hypothalamic-pituitary-adrenal (HPA) axis are activated in mice and humans. The amygdala governs the stress response by acting as the key regulator for mediating aversive memory and bodily functions during stressful events. In particular, the central nucleus of the amygdala (CeA) projects to brain stem centres, regulating blood pressure and cardiovascular reflexes (Saha, 2005), as well as promoting sympathetic activation and reducing vagal efferent outflow to the heart (Park and Thayer, 2014, Thayer et al., 2009). CeA projections can directly stimulate the HPA axis via corticotropin releasing factor-producing cells that project to the paraventricular nucleus of the hypothalamus (Chudoba and Dabrowska, 2023), thereby forming the neuroanatomical framework, consistent with the neurovisceral integration hypothesis that describes cardiac regulation by central autonomic networks during emotional states (Thayer and Lane, 2000). This activation of the sympathetic nervous system and the HPA axis, additionally modulates the immune system: Acute stress increases cell mediated immune responses whereas chronic stress may initially supress immune responses but leads to a dysregulated cellular immune response on a variety of immune cells in different tissues and increases the level of proinflammatory cytokines. This systemic increase in proinflammatory cytokines, then modulates a multitude of different tissue systems such as gut, liver, skin, lung and brain (Yu et al., 2026, Alotiby, 2024, Haykin and Rolls, 2021). Inflammatory responses, following aversive events, bidirectionally mediate emotional and physiological states. The cellular impact of dysregulated inflammatory responses and autonomic imbalance under stress are a matter of intensive research (Liguori et al., 2018, Richard Jennings et al., 2014, Borovikova et al., 2000, Thayer and Lane, 2000). Sustained stress/inflammation lead to an increase in energy demand that is met by elevated oxygen consumption. This can lead to the production of reactive oxygen species (ROS). ROS can damage proteins, poses oxidative stress to cells and tissue and leads to inflammatory signalling. Although structural changes are more prominently linked to sustained stress and therefore ROS exposure (Nazzi et al., 2024, Liguori et al., 2018), we propose that under acute stress, early functional cardiac alterations may still emerge in a sex-specific manner, because of differences in the modulation of oxidative and inflammatory responses. Reduced inflammatory response and ROS production as seen in premenopausal women, is linked to higher oestrogen levels in animals and humans (Novella et al., 2025). Premenopausal women show a higher vagal efferent outflow to the heart, indicated by their heart rate variability (HRV; Smetana and Malik, 2013). Our findings from the CFC animal model may contribute to observed sex-dependent shifts in sympatho-vagal balance, that can be observed in patients with anxiety disorders (Chalmers et al., 2014) for which women have nearly doubled prevalence for developing such disorders (McLean et al., 2011). While hormonal factors, variations in hypothalamic–pituitary–adrenal axis regulation and emotional reactivity of anxiety-related brain areas such as the amygdala have been described as possible biological correlates for sex differences in anxiety disorders (Domes et al., 2010, Bangasser and Valentino, 2014), the contribution of heart-brain interactions and sympathetic integration under stress and emotional memory formation remains a topic of intensive research. In this context, our data after fear conditioning may help to understand sex differences in vulnerability to stress-related cardiovascular dysregulation.

## CRediT authorship contribution statement

**Chirich Barreira Lara:** Writing – review & editing, Validation, Methodology, Investigation, Data curation, Conceptualization. **Luca M. Lautenschläger:** Writing – review & editing, Writing – original draft, Visualization, Validation, Methodology, Investigation, Formal analysis, Data curation, Conceptualization. **Julia Schipke:** Writing – review & editing, Visualization, Supervision. **Jonas Labode:** Writing – review & editing, Supervision, Software. **Christian Mühlfeld:** Writing – review & editing, Validation, Supervision, Resources, Project administration, Methodology, Data curation, Conceptualization. **Anne Albrecht:** Writing – review & editing, Validation, Supervision, Resources, Investigation, Conceptualization.

## Funding

This work was supported by the 10.13039/501100001659German Research Foundation (Project-ID 425899996 – CRC 1436), the Federal Ministry of Education and Research (10.13039/501100002347Bundesministerium für Bildung und Forschung [BMBF]) and the ministry of Saxony-Anhalt within the initial phase of the German Center for Mental Health (DZPG) (project 01EE2305E) and the Center for Behavioural Brain Sciences Magdeburg - CBBS funded by the European funds for regional development (EFRE, Funding Nr. ZS/2016/04/78113) to Anne Albrecht.

## Declaration of Competing Interest

None.

## Data Availability

The data that were used in this study are available from the corresponding author upon reasonable request.
